# Letter Regarding “Outcomes of Bolus Dose Furosemide Versus Continuous Infusion in Patients With Acute Decompensated Left Ventricular Failure and Atrial Fibrillation”

**DOI:** 10.1002/clc.70072

**Published:** 2024-12-26

**Authors:** Ahmed M. Gazer, Elsayed Hammad

**Affiliations:** ^1^ Alexandria University Alexandria faculty of Medicine Champollion Street Alexandria Egypt

Dear Editor,

We read with interest the recent publication by Khan et al. [1] in *Clinical Cardiology* titled “*Outcomes of Bolus Dose Furosemide Versus Continuous Infusion in Patients With Acute Decompensated Left Ventricular Failure and Atrial Fibrillation*”. While the article provides valuable insights into different furosemide strategies in managing patients with acute decompensated heart failure, we noticed an error in Table 1, which presents the baseline patient characteristics. Specifically, the reported patient population underwent a sudden and significant reduction in the arms of the study; for example, an initial cohort of 479 patients reduced or reported to only 14 patients in the T1 arm (intravenous bolus infusion in the table). This error has the potential to misinterpret the results and conclusions of the study.

To address these concerns, we emailed the corresponding author, and we kindly requested that the authors provide clarification regarding:

- The reasons for the significant reduction in patient numbers, including the criteria used for patient exclusion and any attrition during the study.
- The full study protocol and access to the original data for further review.

We believe these clarifications are essential for readers to fully comprehend the validity and applicability of the study's findings.

We kindly request the authors and the journal to rectify this error promptly. A corrected version of Table 1 should be published to ensure accurate interpretation of the study findings.

## Conflicts of Interest

The authors declare no conflicts of interest.

## Data Availability

The authors have nothing to report.
